# The Nature of Wound Shock

**Published:** 1918-04

**Authors:** 


***The Nature of Wound Shock.*** By W. B. CANNON, M. D., *Jour. Amer. Med. Ass'n,* vol. 70, No. 9, p. 611. Abs. by Capt. J. M. WOLFSOHN, M. R. C.

The theory advanced by Henderson, that shock is due to a reduction of the $CO_2$ of the blood (acapnia) is not held by Cannon, who found that painful wounds are not directly associated with a hyperpnea that would produce acapnia. Recently Henderson, in shocked animals, reported a condition of acidosis with a lowered capacity for $CO_2$ in the blood. Because of this Henderson and Porter have recommended re-breathing in shock cases, but Cannon maintains that this state of acidosis which already exists in the shocked patient may be distinctly aggravated by the above procedure. It has been found that the most important consideration in anesthetizing patients suffering from hemorrhage or shock, is to avoid anything in the nature of asphyxia or increased acidosis.

Cannon next combats the idea that in shock, there is a supra-renal exhaustion and consequent hypotension. He proves that patients suffering from wounds have been severely stimulated and concludes that the high percentage of sugar found in the blood of his series of shock cases indicates that the supra-renal glands are, if anything, over-active rather than exhausted.

The Nerve Exhaustion theory of Crile is next attacked because the latter's examinations were largely histologic and, says Cannon, " histologic evidence regarding state of nerve cells is subject to grave mischances both in technique and in interpretation ". The interpretation of cell changes as a result rather than as a cause of shock points to a clear distinction which should be drawn between early and late indications of asthenia in shocked men.

The view that in shock the splanchnic vessels hold the stagnant blood, is disproved for man, by the observations of surgeons in these cases. Cannon sets forth many reasons why the capillary capacity is sufficient to contain all the lost blood in shock and that the more concentrated the blood becomes, the more are its chances of doing so, especially as the concentration of the blood in the capillary areas is increased by loss of heat and by low blood pressure. Shock is observed more often in cold weather, especially when accompanied by rain so that the clothing is wet through. When a wounded man is chilled, his blood pressure falls and, as he becomes warmed, it may rise again.

Anything that will increase the concentration of the H-ion, e. g. relaxation of the vessel walls, especially the capillaries, weakening of the cardiac contractions, and increase of blood viscosity, would be favorable to the continuance of low blood pressure.

From the above considerations there seem to be at least six vicious circles formed, all centering around the H-ion, the viscosity and concentration of the blood, the blood pressure, and the capillary over-filling — and therefore it is important that the treatment of shock be prompt and directed toward preventing an increase of the unfavorable conditions.

Cannon recommends the term " exemia " instead of shock as being more explanatory of the actual condition, as the essential parts of the patient's circulatory system have been drained of blood.

The author's conclusions are a listing of the above facts as follows. There are primary wound shocks with rapid lowering of arterial pressure, and secondary wound shocks with toxemia and hemorrhage and later lowering of the blood pressure. Sweating occurs, leading to loss of fluid and loss of heat from the body. The blood becomes stagnant and concentrated in the capillaries and as the blood pressure falls, there is loss of the alkali reserve in the blood (acidosis), roughly corresponding to a drop in pressure. Previous fatigue and starvation would favor a development of the acidosis.

